# Optical Sensor Based on a Single CdS Nanobelt

**DOI:** 10.3390/s140407332

**Published:** 2014-04-23

**Authors:** Lei Li, Shuming Yang, Feng Han, Liangjun Wang, Xiaotong Zhang, Zhuangde Jiang, Anlian Pan

**Affiliations:** 1 State Key Laboratory for Manufacturing Systems Engineering, Xi'an Jiaotong University, Xi'an 710049, China; E-Mails: leili.1120@stu.xjtu.edu.cn (L.L.); hanfeng_2062@163.com (F.H.); wang1liang2jun3@163.com (L.W.); xiaotong339@hotmail.com (X.Z.); zdjiang@mail.xjtu.edu.cn (Z.J.); 2 State Key Laboratory of Digital Manufacturing Equipment & Technology, Huazhong University of Science and Technology, Wuhan 430074, China; E-Mail: shuming.yang@mail.xjtu.edu.cn; 3 Key Laboratory for Micro-Nano Optoelectronic Devices of Ministry of Education, School of Physics and Microelectronics, Hunan University, Changsha 410082, China; E-Mail: anlian.pan@hnu.edu.cn

**Keywords:** CdS, nanobelt, optical sensor, Schottky contact, photosensitivity

## Abstract

In this paper, an optical sensor based on a cadmium sulfide (CdS) nanobelt has been developed. The CdS nanobelt was synthesized by the vapor phase transportation (VPT) method. X-Ray Diffraction (XRD) and Transmission Electron Microscopy (TEM) results revealed that the nanobelt had a hexagonal wurtzite structure of CdS and presented good crystal quality. A single nanobelt Schottky contact optical sensor was fabricated by the electron beam lithography (EBL) technique, and the device current-voltage results showed back-to-back Schottky diode characteristics. The photosensitivity, dark current and the decay time of the sensor were 4 × 10^4^, 31 ms and 0.2 pA, respectively. The high photosensitivity and the short decay time were because of the exponential dependence of photocurrent on the number of the surface charges and the configuration of the back to back Schottky junctions.

## 1. Introduction

Semiconductor nanobelts have attracted intensive attention for constructing the new-generation nano optoelectronic devices such as solar cells, light emitting diodes (LED), laser diodes and optical sensors, *etc.* [1–5]. Regarding optical sensors, nanobelts can reduce the operation voltage and energy consumption, increase the response speed and photosensitivity. Semiconductor nanobelt optical sensors have potential applications in high-speed optical communication, image technology, optoelectronic circuits and wireless intelligent sensor network, *etc*.

One-dimensional semiconductor nanostructures based on group IV (Si, Ge) [6,7], group II-VI (ZnO, ZnS, CdS, CdSe, CdS_x_Se_1-x_ HgTe) [5,8–12], and group III-V (GaN, InN, InP, GaAs AlN) compounds have been developed [13–17]. Among these materials, CdS is promising one for visible light detection due to its band gap of 2.4 eV, corresponding to the center of the visible light region, and high photosensitivity [1,18,19]. At the same time, the band gap of CdS can be easily tuned by forming ternary compounds with CdSe for application in tunable photodetectors [12,20]. For optical sensors based on CdS nanobelts, it is important to form ohmic contact at the metal-semiconductor interface. The purpose is to reduce the contact resistance and achieve a steady electrical contact, but this kind of devices show relatively low photosensitivity and slow photoresponse speed [21]. Recently, some researchers have focused on the Schottky contact metal-semiconductor-metal (MSM) nanobelt optical sensors [22,23]. Compared with the ohmic contact nanobelt photoconductive sensor, the Schottky contact sensor presented much higher photosensitivity and quicker photoresponse [21,22].

In this paper, CdS nanobelt MSM optical sensors with two Schottky contacts were fabricated. The Schottky contacts were realized by deposition of Pd/Ti (100 nm/20 nm) onto the nanobelt [24]. The device presented a photosensitivity of about 4 × 10^4^. The decay time was about 31 ms, and the dark current is as low as 0.2 pA.

## 2. Experiments

### 2.1. CdS Nanobelt Synthesis and Characterization

High purity CdS powder (Aldrich, Los Angeles, CA, USA, 99.99% purity) was used to synthesize nanobelt based on the vapor phase transportation (VPT) method [25]. The evaporation temperature was 950 °C and the growth time was 20 min. The synthesis process was carried out in argon atmosphere at a pressure of 0.7 bar. The nanobelt morphology and elemental composition was investigated by SEM (SU-8010 Hitachi, Tokyo, Japan, operated at 10 kV) and Energy-Dispersive X-ray Spectrum (EDS) installed on the SEM. XRD (XPert Pro MRD, Almelo, Netherlands operated at 40 kV, 40 mA) with Cu Kα radiation and High-Resolution TEM (HRTEM, JEOL JEM-2100, Akishima, Japan, operating voltage 200 kV) were used to characterize the crystal structure and quality of the CdS nanobelt.

### 2.2. Single Nanobelt Optical Sensor Fabricating and Testing

**Figure 1.**
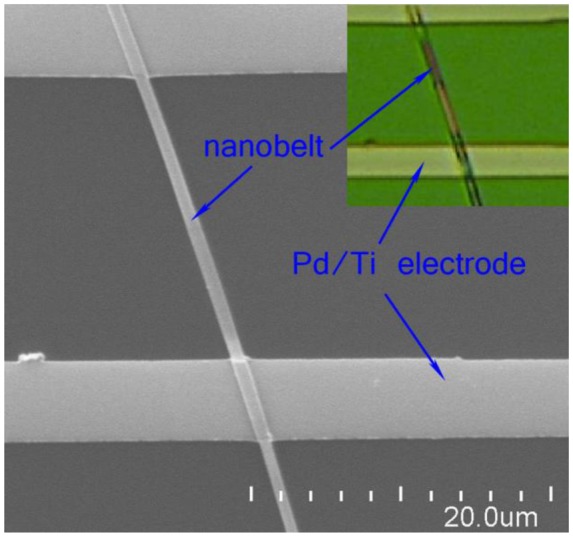
The SEM image of the finished single nanobelt optical sensor, the upper right insert was the optical microscope image of the device.

A single CdS nanobelt optical sensor was fabricated by e-beam lithography combined with the metal sputtering deposition technique [26,27]. As-grown CdS nanobelt was transferred from the Si substrate into an isopropanol (IPA) solution and ultrasonically dispersed for 5 min to form a homogeneous “nanobelt-IPA” suspension. Si wafer capped with 300 nm Si_3_N_4_ was used as the device substrate. The substrate was firstly ultrasonically cleaned in acetone, ethanol and deionized (DI) water, for 10 min respectively, and then dried by N_2_ flow. Several drops of the nanobelt-IPA suspension were spin coated onto the substrate, and then the substrate was heated up to 80 °C to evaporate the IPA solution. Next 500 nm PMMA (950 k) e-beam photoresist layer was spin coated on the substrate. The electrode patterns were defined by e-beam lithography (CABL 9000C, Crestec, Shizuoka, Japan), followed by radio-frequency magnetron sputtering (explorer 14, Denton Vacuum, Moorestown, NJ, USA) depositing Pd/Ti (100 nm/20 nm) layer and lift-off process. Figure 1 shows a SEM image and an optical microscope image of the finished single nanobelt optical sensor. The white regions were the Pd/Ti electrodes and the dark gray region was the Si_3_N_4_ substrate. It can be seen that the nanobelt was well capped by the metal electrodes.

The current-voltage characteristics were measured by a semiconductor characterization system (4200SCS, Keithley, Cleveland, OH, USA) connected with a probe station (PEH-4, EverBeing Int'l Corp., Hsinchu, Taiwan). The photoresponse characteristics were tested using a white light source (MLC-150C, Motic, Xiamen, China) equipped on the probe station as the incident light.

## 3. Results and Discussion

### 3.1. Nanobelt Synthesis and Characterization

**Figure 2.**
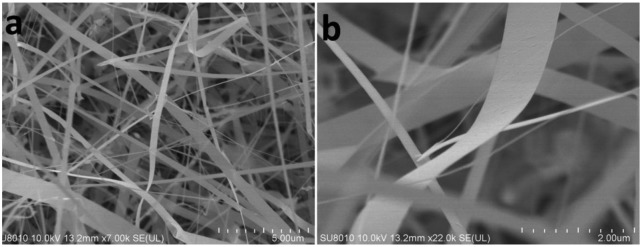
The SEM image of the as-grown CdS nanobelts.

Figure 2 shows SEM images of the CdS nanobelt, it can be seen that the width of the nanobelts was distributed from several hundred nanometers to several micrometers. The thicknesses of most nanobelts were only tens of nanometers.

**Figure 3.**
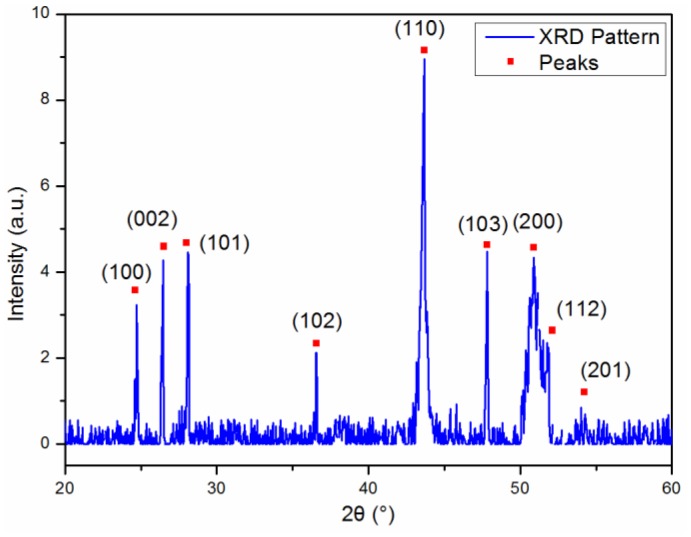
XRD pattern of the CdS nanobelt.

Energy Dispersive X-ray Spectrum (EDS) of the CdS nanobelt indicated that the nanobelt was composed of cadmium (Cd) and sulfur (S), and the elemental ratio of S and Cd was 0.92. Figure 3 shows the XRD pattern of the CdS nanobelts. The diffraction peaks can be indexed to the typical hexagonal wurtzite structure CdS crystal (JCPDS No. 41-1049) with the *c* = 6.728 Å and *a* = 4.145 Å consisting with the lattice parameter of wurtzite CdS. No diffraction peaks of cadmium oxide (CdO), Cd, S or other impurities were found.

**Figure 4.**
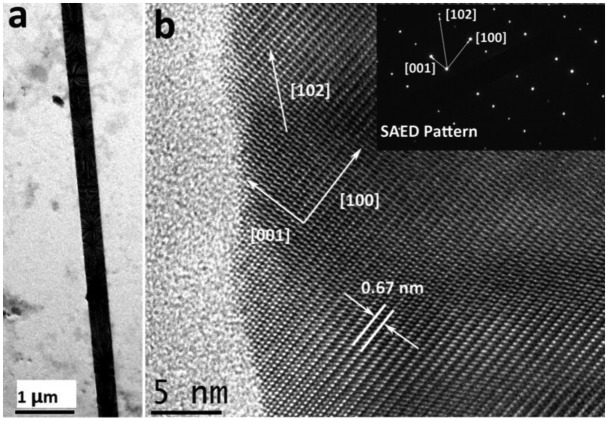
TEM, HRTEM and SAED image of the single CdS nanobelt.

Figure 4 shows the TEM results of the single CdS nanobelt. It can be seen that the as-grown CdS nanobelt presented very uniform width and smooth edges. The interplanar distances was 0.67 nm which corresponds to the hexagonal wurtzite CdS crystal (001) crystal interplanar spacing [28]. The inset upper right image in Figure 4 was the Selected Area Electron Diffraction (SAED) pattern of the single nanobelt. The nanobelt showed good single crystal quality and the growth orientation was [102].

### 3.2. Single CdS Nanobelt Optical Sensor Fabricating, Testing and Analysis

**Figure 5.**
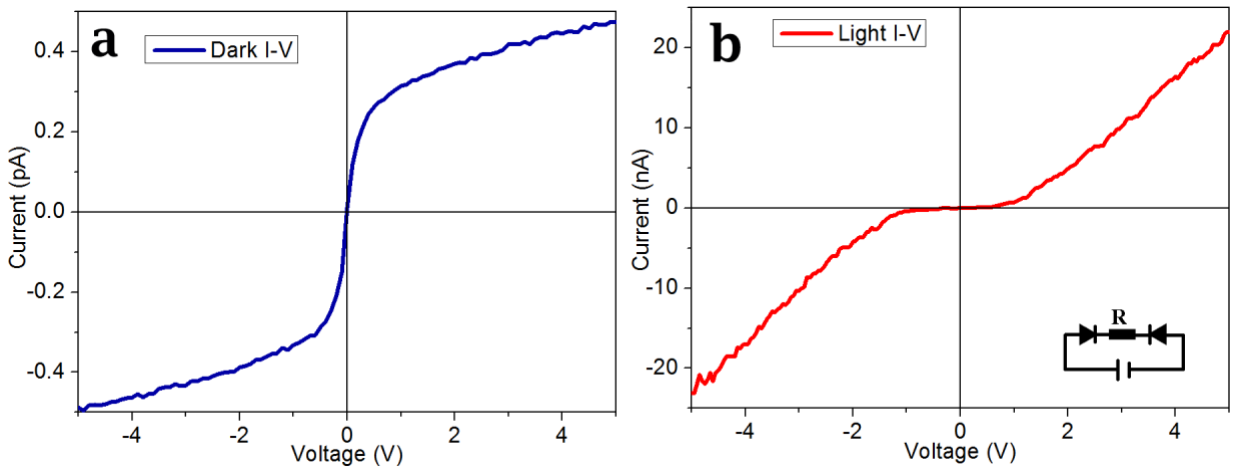
I-V curves of the device: (**a**) was the I-V curve without light illumination; (**b**) was the I-V curve with light illumination; the lower right insert of b was the schematic diagram of the device.

**Figure 6.**
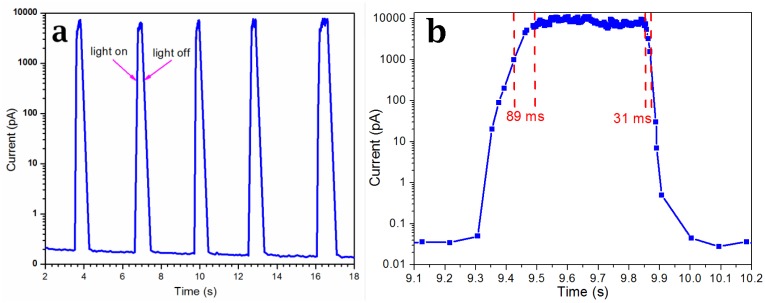
(**a**) The light response of the sensor; (**b**) the detailed decay curve and the decay time calculated from the curve was about 31 ms.

I-V curves were measured using a two-terminal configuration, one electrode was grounded and the other was supplied with the bias voltage of *V_bias_*. Figure 5 gives the dark and light I-V curves of the device. The inset lower right was the schematic diagram of the device, R was the resistance of the CdS nanobelt and the device was equivalent to a back-to-back Schottky diode [23,29]. The dark current of the device was only 0.5 pA when the *V_bias_* < 5 V. This was because of the high intrinsic electric resistance of the as-grown CdS nanobelt and the barrier of the reverse/forward biased Schottky junctions [22,30,31]. The device light I-V curve exhibited obvious diode characteristics. When the *V_bias_* > 1 V, the current increased quickly, and reached to about 22 nA at a *V_bias_* of 5 V. The photoresponse properties were measured at the *V_bias_* of 3 V. Figure 6a corresponds to the light response curve. The dark current value was about 0.2 pA, and jumped to about 8 nA when the light was on. Figure 6b shows the detailed photoresponse curve. The responsivity was defined as *S_I_* = *I*/(*P* × *S*), where *I:* the light current was 8 nA; *P:* the power intensity of the light source was 0.3 W/cm^2^; *S:* the working area of the device was 20 μm × 0.6 μm =12 × 10^−8^ cm^2^. The *S_I_* was 0.22 A/W. The photosensitivity defined as the ratio of photocurrent to dark current was 4 × 10^4^. This was much higher than reported by Wei *et al.* [21] who reported a value of 1,000 and Yi *et al.* [29] who reported one of about 4. The calculated rising time *τ_r_* and decay time *τ_d_*(*τ_r_*(*τ_d_*) were defined as the times needed to rise (fall) from 10% (90%) to 90% (10%) of the light current) were about 89 ms and 31 ms, respectively. The decay time was higher than reported by Wei *et al.* [21] (572 ms) and Yi Xi *et al.* [32] (0.2 s).

The oxygen in the air can combine with the electrons of the n-type CdS nanobelt $\left(O_{2}+e^{-}\to {O^{-}}_{2}\right)$ and absorb onto the nanobelt surface. This led to an electron depletion layer near the surface of the nanobelt, which can narrow the current channel and reduce the conductivity. For ohmic contact CdS nanobelt optical sensor, the photoresponse was mainly because the adsorbed O_2_ combined with photon generated holes and desorbed $\left({O^{-}}_{2}+h^{+}\to O_{2}\right)$ . The depletion layer will be thinned, and the conductance was increased. At the same time, the photon generated carriers were separated by the external electric field and also participated in electric conduction [1,21,32].

**Figure 7.**
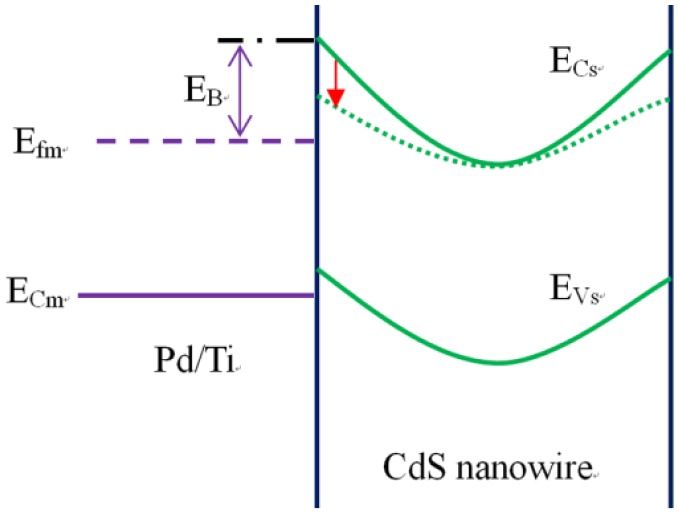
The Schottky barrier energy band diagram and the photoresponse mechanism: when light was on, the photo generated holes will recombine with the adsorbed oxygen on the nanobelt surface. The surface depletion layer thickness and the Schottky barrier E_B_ were reduced. E_Cs_ and E_Vs_ were the conductance band and valance band of the CdS nanowire; E_fm_ and E_Cm_ were the Fermi level and conductance band of the metal. E_B_ was the Schottky barrier.

For a Schottky contact optical sensor, the main photoresponse occurred at the Schottky junctions and mainly the reverse biased junction [23]. The mechanism was the modulation of Schottky barrier by the light illumination. As shown in Figure 7, the surface depletion layer caused by the absorbed O_2_ can increase the Schottky barrier height E_B_.

When light was on, the negative charged oxygen combined with the photo generated holes and desorbed. The surface depletion layer thickness and the Schottky barrier height E_B_ would be reduced [21]. The Schottky barrier height E_B_ was in direct proportion to ${N^{2}}_{S}$ (*N_S_* is the number of the surface charges) [33]. At the same time, the current of the Schottky contact photodetector was mainly attributed to the tunneling current of the Schottky barrier. The tunneling current decreased exponentially with the increase of E_B_ [34], so the increase of the *N_S_* will generate an exponential decrease of the current. For the ohmic contact photodetector, the photocurrent decreased linearly with the increase of the *N_S_* [33]. Therefore, the current of the Schottky contact optical sensor was much more sensitive to the number of the surface charges, and this can explain the short decay time for the Schottky contacted optical sensors.

On the other hand, because of the exponential dependence of the photocurrent on the surface charges, the photosensitivity was much higher than the ohmic contact optical sensor which was linearly dependent on the surface charges. Also, because of the configuration of the device, one of the Schottky junctions was reverse biased, which caused an obvious decrease of the dark current and left more space for possible photocurrent improvement [21,23], so the photosensitivity was much higher than ohmic contact optical sensors.

## 4. Conclusions

A single nanobelt optical sensor with two Schottky contacts has been fabricated by the EBL technique. The decay time was 31 ms, and the photosensitivity was 4 × 10^4^. The short decay time and high photosensitivity were because of the exponential dependence of the photocurrent on the number of surface charges, and the series of forward and reverse biased Schottky junctions. The nanobelt optical sensor can potentially be used for high-sensitivity and high speed light detection, and can be easily integrated into multifunctional nanochips.
